# Reference distributions of aortic calcification and association with Framingham risk score

**DOI:** 10.1038/s41598-023-36565-8

**Published:** 2023-06-09

**Authors:** Steven R. Horbal, Brian A. Derstine, Edward Brown, Grace L. Su, Stewart C. Wang, Sven A. Holcombe

**Affiliations:** 1 Morphomics Analysis Group, University of Michigan, Ann Arbor, MI USA; 2 Division of Gastroenterology and Hepatology, University of Michigan, Ann Arbor, MI USA; 3 Gastroenterology Section, Veterans Administration Ann Arbor Healthcare System, Ann Arbor, MI USA; 4 Department of Surgery, University of Michigan, Ann Arbor, MI USA

**Keywords:** Calcification, Epidemiology, Diagnostic markers, Atherosclerosis

## Abstract

Evidence supporting aortic calcification as a leverageable cardiovascular risk factor is rapidly growing. Given aortic calcification’s potential as a clinical correlate, we assessed granular vertebral-indexed calcification measurements of the abdominal aorta in a well curated reference population. We evaluated the relationship of aortic calcification measurements with Framingham risk scores. After exclusion, 4073 participants from the Reference Analytic Morphomic Population with varying vertebral levels were included. The percent of the aortic wall calcified was used to assess calcification burden at the L1–L4 levels. Descriptive statistics of participants, sex-specific vertebral indexed calcification measurements, relational plots, and relevant associations are reported. Mean aortic attenuation was higher in female than male participants. Overall, mean aortic calcium was higher with reference to inferior abdominal aortic measurements and demonstrated significant differences across all abdominal levels [L3 Area (mm$^2$): Females 6.34 (sd 16.60), Males 6.23 (sd 17.21); L3 Volume (mm$^3$): Females 178.90 (sd 474.19), Males 195.80 (sd 547.36); Wall Calcification (%): Females (L4) 6.97 (sd 16.03), Males (L3) 5.46 (13.80)]. Participants with elevated calcification had significantly higher Framingham risk scores compared to participants with normal calcification scores. Opportunistically measuring aortic calcification may inform further cardiovascular risk assessment and enhance cardiovascular event surveillance efforts.

## Introduction

Cardiovascular disease (CVD) remains the leading cause of death in the United States: when excluding hypertension, estimated prevalence is 9.3% overall^1^. The fundamental disease process of CVD is atherosclerosis, a chronic inflammatory condition that forms calcified plaque, promotes further plaque formation, and influences vessel narrowing^2,3^.

Vascular calcification scoring is a robust prediction tool for quantifying total atherosclerotic burden and improves the predictive ability of conventional models of risk^4–6^. Coronary artery calcium (CAC) scores are quantitatively assessed from specific computed tomography (CT) scans, are highly sensitive, thoroughly studied, cost-effective, and strong predictors of major cardiovascular events^7–9^. Aortic calcification, while less studied, shows strong promise in preventative screening of clinical populations and is measurable in conventional abdominal CT. Aortic calcification correlates with CAC and is associated with traditional risk factors such as age, systolic blood pressure, smoking, and LDL cholesterol^10–15^. Recent research has demonstrated that aortic calcification scores can predict cardiovascular events and may outperform the Framingham Risk Score (FRS)^13,16–18^.

There is no current consensus on standard measurement for aortic calcification from CT-scans. Many methods of obtaining aortic calcification measurements are derived from coronary artery calcification measurement and methodologies^19^. For example, Graffy et al. use a region-based convolutional neural network to segment the abdominal aorta and then calculate the Agatston, volume, and mass score for each plaque^20^. In addition to Agatston scores, the count of calcified lesions, lesion phenotype (thickness, length), and percent of aortic wall are employed measurement strategies to justify aortic calfication’s clinical relevance^15,21^. Region specific investigations of full aortic, thoracic aortic, and abdominal aortic calcium have been investigated^14,15,20,22–24^. Intravenous contrast is a major confounder of aortic calcification measurement, and as such, studies tend to stratify by use of contrast or phase^19,25^. Efforts to control for this confounding and create valid population-level estimates are ongoing^25,26^.

Analytic Morphomics (morphomics) is a semi-automated computational image processing system developed by the Morphomics Analysis Group (MAG) at the University of Michigan^27^. Morphomics provides vertebral indexed, granular measurements of body composition. These measurements are utilized as digital biomarkers for clinical purposes. Using morphomics data, MAG has developed cohorts of routine clinical CT scans in an effort to capture the heterogeneity of body composition in the hospital-based and general populations^28–30^.

To our knowledge, descriptions or distributions of aortic calcium in reference populations are absent from the literature. Given the growing interest in aortic calcification as a clinical correlate for cardiovascular disease and the importance of early identification of CVD for prevention efforts, the purposes of this analysis include (1) describe the distribution of aortic calcium in reference population not conditioned on cardiovascular disease and (2) evaluate the Morphomic Aortic Calcification score’s suitability as a risk indicator of cardiovascular disease.

## Results

**Table 1:** Descriptive Statistics of RAMP Sample by Sex.

	Female (1388)	Male (2685)	p
Age (year)	43.81 (20.27)	40.77 (17.47)	< 0.01
BMI ($kg/m^2$) (n=2992)	27.31 (6.92)	27.75 (5.98)	0.07
Mean attenuation (HU)	185.38 (64.49)	159.64 (53.59)	< 0.01
Calcified area ($mm^2$)	6.31 (16.45)	6.17 (17.06)	0.80
Calcified volume ($mm^3$)	177.33 (468.85)	193.33 (542.36)	0.35
Total aortic wall area ($mm^2$)	1270.47 (472.26)	1568.48 (603.54)	< 0.01
Wall % calcification (%)	6.79 (15.9)	5.51 (13.5)	0.01
Elevated MAC (n (%))*	310 (22.8)	564 (21.3)	0.28
Elevated MAC (n (%))**	196 (15.5)	328 (13.2)	0.06
Zero calcification (n (%))	966 (69.6)	1872 (69.7)	0.96
HDL cholesterol (n=1022)	122.08 (21.68)	128.54 (23.51)	0.01
Total cholesterol (n= 1022)	184.38 (48.21)	177.32 (49.22)	0.02
Systolic BP (n=3290)	55.36 (17.91)	44.68 (15.67)	< 0.01
Smoking (n=2115)	–	–	< 0.01
Current	178 (24.6)	394 (33.7)	
Former	142 (19.6)	278 (23.8)	
Never	404 (55.8)	496 (42.5)	
Framingham score	5.95 (6.06)	6.95 (5.84)	< 0.01

Measurements reported as mean (SD) or count (%) where noted.

*Patient considered ’Elevated’ if L3 or L4 have MAC > 4.21%, calibrated for sensitivity.

**Patient considered ’Elevated’ if L3 or L4 have MAC > 12.93%, calibrated for for balance.

Morphomic measurements are averaged across L1–L4, for vertebral specific measurements, see Table 2.

Table 1 reports the descriptive statistics of the RAMP sample, stratified by sex. Mean female age [43.81 (sd 20.27)] was significantly higher than mean male age [40.77 (sd 17.47)]. Significant differences were observed in mean attenuation [females 185.38 (sd 64.45), males 159.64 (sd 53.59), p < 0.01], aortic wall area [females 1270.47 mm$^2$ (sd 472.26), males 1568.48 (sd 603.54) p < 0.01], and the percent of the aortic wall calcified [females 6.79 % (sd 15.93), males 5.51 % (sd 21.3) p < 0.01]. HDL cholesterol [n = 1022: females 122.08 (sd 21.68), males 128.54 (23.51] total cholesterol [n = 1022: females 122.08 (21.68, m a les 128.53 (23.51], total cholesterol [females 184.38 (48.21), males 177.32 (49.22)] systolic blood pressure [n = 3290: females 55.36 (17.91, males 44.68 (sd 15.67)] and the proportion of smokers (p < 0.01) were all significantly different. Framingham scores were higher in male participants compared to female participants [female 5.95 (sd 6.06) male 6.95 (sd 5.84).

**Table 2:** Descriptive Statistics of RAMP Sample by Vertebral Level.

	L1 (4027)	L2 (3978)	L3 (3973)	L4 (3747)	p
Age (mean (SD))	41.62 (18.44)	41.54 (18.38)	41.60 (18.42)	41.90 (18.53)	0.83
Male Sex (%)	2654 (65.9)	2628 (66.1)	2625 (66.1)	2504 (66.8)	0.83
BMI	27.60 (6.32)	27.64 (6.33)	27.64 (6.33)	27.60 (6.34)	0.99
Mean attenuation (HU)	178.98 (66.07)	168.82 (57.64)	168.17 (57.22)	167.65 (61.89)	< 0.01
Calcified area ($mm^2$)	2.15 (8.33)	3.69 (13.07)	6.27 (17.00)	6.02 (16.72)	<0.01
Calcified volume ($mm^3$)	71.15 (274.66)	110.67 (394.22)	190.07 (523.68)	139.10 (421.17)	$<<$0.01
Total wall area ($mm^2$)	1780.59 (701.13)	1414.44 (440.97)	1461.74 (556.50)	978.24 (796.19)	<0.01
Wall % calcification	1.79 (6.30)	3.37 (10.41)	5.99 (14.51)	5.96 (14.59)	<0.01
Elevated MAC* (n (%))	856 (21.6)	857 (21.6)	862 (21.7)	825 (22.0)	0.97
Elevated MAC* (n (%))	587 (14.8)	588 (14.8)	593 (14.9)	545 (14.5)	0.97
Zero calcification (n (%))	3231 (80.2)	3061 (76.9)	2785 (70.1)	2691 (71.8)	<0.01

Measurements reported as mean (SD) or count (%) where noted.

*Patient considered 'Elevated’ if L3 or L4 have MAC > 4.21%, calibrated for sensitivity.

**Patient considered 'Elevated’ if L3 or L4 have MAC > 12.93%, calibrated for for balance.

Table 2 reports anthropomorphic and morphomic measurements stratified by vertebral level. Significant differences were observed in mean aortic attenuation, cross sectional calcification area, cross sectional calcification volume, aortic wall area, wall percent calcification, and the proportion of participants with zero observed calcification (< 0.01).

**Table 3:** Calcification Measurements by Vertebral Level.

Vertebra	Females			Males		
	Calcification area (mm$^2$)			Calcification area (mm$^2$)		
	Mean (SD)	Median	90th	Mean (SD)	Median	90th
L1	2.32 (8.28)	0.00	5.21	2.07 (8.36)	0.00	3.12
L2	3.85 (12.40)	0.00	10.40	3.60 (13.40)	0.00	7.60
L3	6.34 (16.60)	0.00	22.50	6.23 (17.21)	0.00	21.32
L4	6.31 (16.30)	0.00	21.69	5.87 (16.90)	0.00	19.11

Vertebra	Calcification volume (mm$^3$)			Calcification volume (mm$^3$)		
	Mean (SD)	Median	90th	Mean (SD)	Median	90th
L1	70.84 (251.94)	0.00	169.16	71.31 (285.75)	0.00	109.16
L2	108.82 (345.97)	0.00	299.96	111.62 (416.90)	0.00	230.65
L3	178.90 (474.19)	0.00	611.19	195.80 (547.36)	0.00	481.65
L4	140.67 (388.73)	0.00	481.65	138.31 (436.45)	0.00	400.36

Vertebra	Wall calcification %			Wall calcification %		
	Mean (SD)	Median	90th	Mean (SD)	Median	90th
L1	2.09 (6.91)	0.00	5.63	1.62 (6.00)	0.00	3.19
L2	3.96 (11.26)	0.00	12.57	3.07 (9.92)	0.00	7.99
L3	6.81 (16.05)	0.00	26.73	5.56 (13.62)	0.00	20.68
L4	6.97 (16.03)	0.00	27.74	5.46 (13.80)	0.00	19.44

Table 3 reports measurements of calcification area and calcification volume relative to lumbar vertebra. Mean area measurements were highest at L3 for females [6.34 mm$^2$ (sd 16.60)] and for males [6.23 mm$^2$ (sd 17.21]. Calcification volume measures were observed to be the largest at L3 [female volume 178.90 mm$^3$ (sd 474.19),males 195.80 mm$^3$ (sd 547.36)]. Wall calcification percent was higher at L3 and L4 vertebral levels relative to L1 and L2. Mean wall calcification percent was observed to be highest at L4 [6.97 (sd 16.03) for females and 5.56 (13.62) for males]. The median values were 0 for all measures, indicating a strong left skew for all levels among both area, volume, and wall calcification percent.

**Figure 1:**
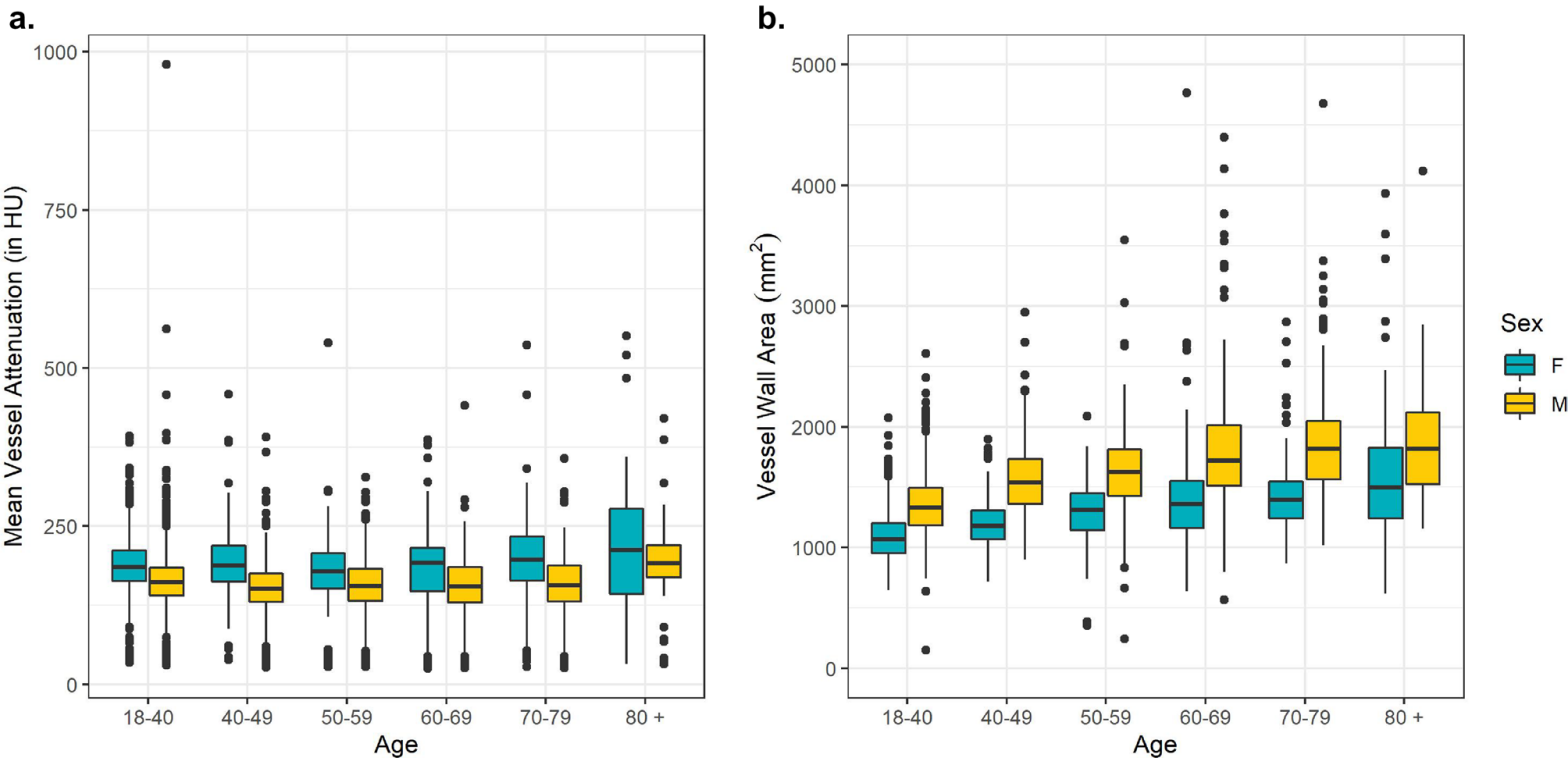
Sex-stratified boxplots for (**a**) age and mean aortic attenuation (**b**) age and mean vessel area.

Boxplots describing the distribution of mean aortic attenuation (L1-L4 vertebral levels) by age and sex are reported in Fig. 1 (left). For females, median aortic attenuation was 184.94 (18–40), 188.59 (40–49), 177.93 (50–59), 188.63 (60–69), 194.91 (70–79), 212.48 (80+). For males, median aortic attenuation was 161.19 (18–40), 151.81 (40–49), 154.94 (50–59), 150.56 (60–69), 157.43 (70–79), 188.54 (80 +). Females had higher attenuation among all age groups. Distributions of vessel wall area (L1–L4) by age and stratified by sex are displayed in Fig. 1 (right). Males had consistently larger vessel wall area than females. Vessel wall area increased with age for males and females. Linear regression demonstrated strong relationships of vessel wall area with age and sex [Age ($\beta$ 9.31, p < 0.01), Male ($\beta$ 320.38, p < 0.01)].

**Figure 2:**
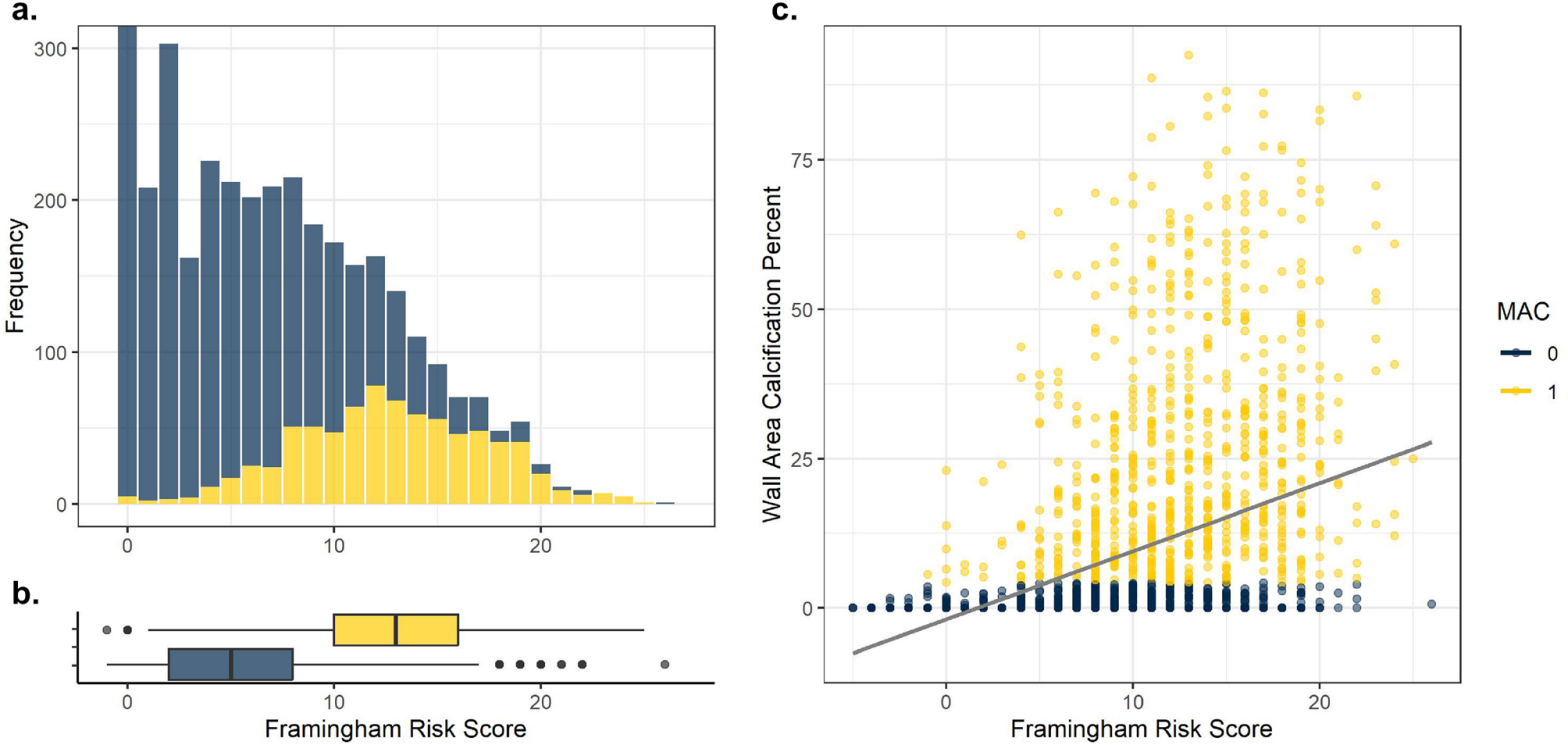
Framingham risk scores and MAC in RAMP (**a**) Frequencies of FRS. Scores with “0” risk exceed the y axis labels (n = 662). (**b**) Box plots depicting distributions of FRS in participants with elevated (Wall Area Calcification Percent < 4.21 (yellow) and > 4.21 (blue). (**c**) Scatter plot of Framingham Risk Scores by Wall Area Percent.

**Figure 3:**
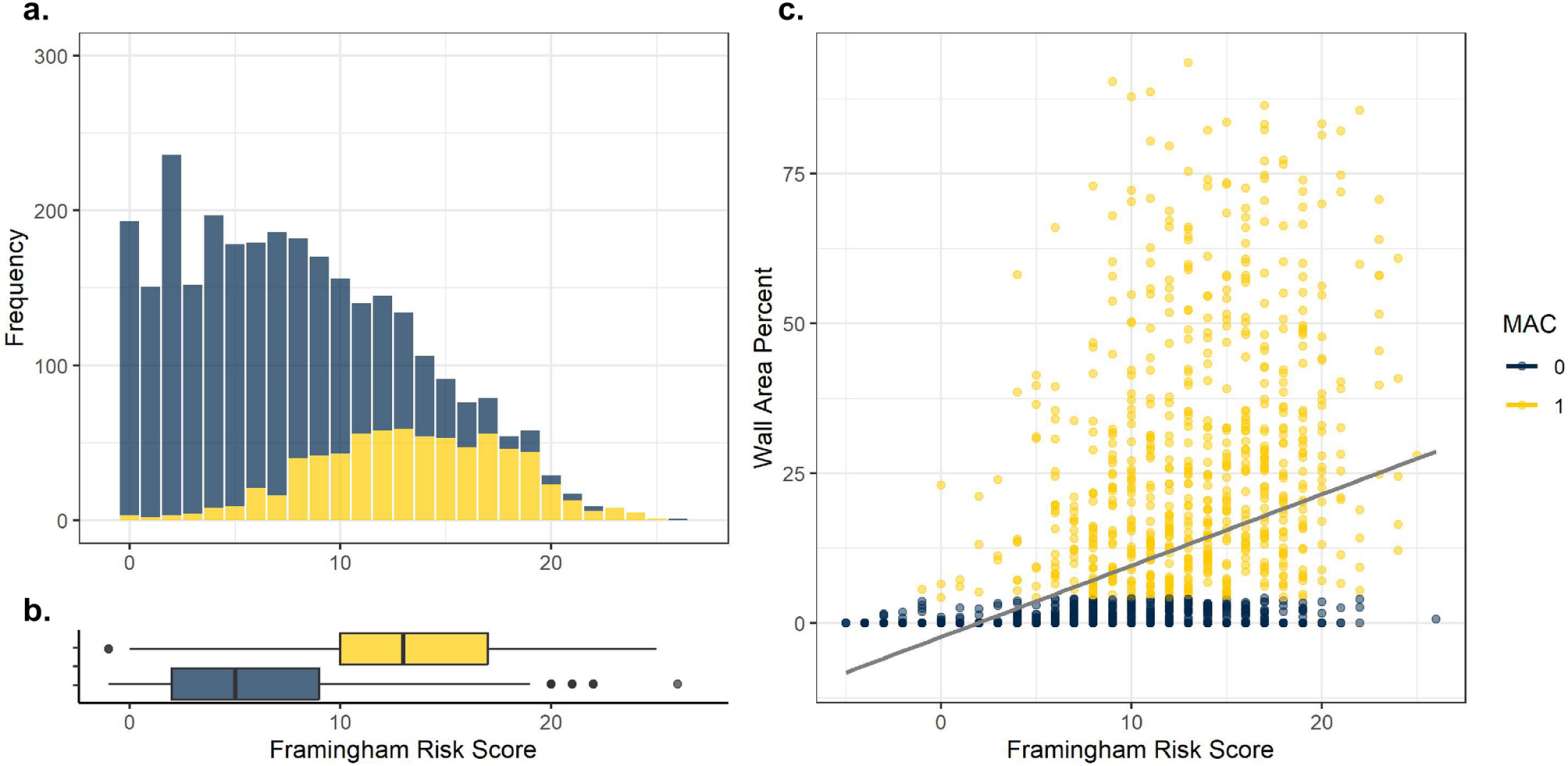
Framingham risk scores and MAC in RAMP, participants with missing systolic blood pressure removed. (**a**) Frequencies of FRS. (**b**) Box plots depicting distributions of FRS in participants with elevated (Wall Area Calcification Percent < 4.21 (yellow) and > 4.21 (blue). (**c**) Scatter plot of Framingham Risk Scores by Wall Area Percent.

Figure 2 demonstrates the distributions of the estimated Framingham Risk Scores for the RAMP subsample. For all participants, the mean Framingham score was 6.61 and the median score was 6. Overall the distribution appears to exhibit a bimodal pattern with a mode at 0 and secondarily at 2. Overall shape is right skewed with the maximum observed score being 26 points. Conditioning on an elevated MAC score, the participants with normal MAC score (MAC<4.21%) versus elevated MAC score (MAC $\ge$ 4.21) demonstrates strong separation of means between both groups. Those with a normal MAC score had a mean Framingham score was 4.84, the median score was 4. The shape of the distribution was slightly right skewed. Those with an elevated MAC score had a mean Framingham score was 12.93, the median score was 13. The shape of the distribution was slightly left skewed. Wilcoxon signed ranked test indicated indicated strong differences in mean FRS among those with elevated and normal MAC scores (p $<<$ 0.001). Linear regression indicated a significant univariate association of FRS with log transformed wall % calcification (Int − 4.78 $\beta$ 0.34 p $<<$ 0.01, R$^2$ 0.39).

Figure 3 demonstrates distributions of the Framingham Risk Scores for the RAMP subsample after removing those without measured systolic blood pressure. Overall, a similar distribution to Fig. 2 was observed. Those with a normal MAC score had a mean Framingham score was 5.31, the median score was 5. Those with an elevated MAC score had a mean Framingham score was 13.37, the median score was 13. Wilcoxon signed ranked test indicated indicated strong differences in mean FRS among those with elevated and normal MAC scores (p $<<$ 0.001). MAC scores (p $<<$ 0.001). Linear regression indicated a significant univariate association of FRS with log transformed wall % calcification (Int − 4.82 $\beta$ 0.33 p $<<$ 0.01, R$^2$ 0.37).

## Discussion

This study reports sex-specific, vertebral indexed calcification measurements from the abdominal aorta in a reference population not conditioned on cardiovascular disease. Aortic calcification area and volume measurements were highest at L3 and L4 relative to L1 and L2. Measurements for axial vessel area and aortic attenuation are also reported.

Our previous research has demonstrated strong relationships between coronary calcification scores and abdominal aortic calcification (MAC), specifically at the L3 and L4 levels^15,25,26^. Leveraging such evidence, we proposed the use of the MAC score to approximate coronary calcium measurement using computed tomography scans of the abdominal aorta^15,26^. In this study, the MAC score and aortic wall percent calcification were found to be strongly associated with the Framingham Risk Score. Participants with elevated MAC scores had significantly higher FRS compared to patients with normal-level MAC scores. While vascular calcification is a slow, dynamic process, the earliest vascular beds to exhibit atherosclerotic calcification are typically in the abdominal aorta^31–33^. As abdominal CT scans are typical of routine care, these results provide evidence supporting the usefulness of MAC as a potential screening mechanism for those with premature development of calcification.

The descriptive results of this paper are consistent with the literature, notably the anatomical location of calcium and its relationship with age. The magnitude of atherosclerotic burden is consistently reported to be greater in the descending than the ascending aorta, and in the abdominal rather than the thoracic aorta^15,17,18,34^. Previous anatomical research describes increasing aortic dilation and aortic calcification with advancing age^35,36^. Aging related changes in the aorta include thicker tunica intema between distal and proximal portion as well as damaged internal elastin, collagen and smooth muscle fibers^36–41^. This fibrous damage paired with aortic calcification contributes to aortic stiffening and increasing aortic pulse wave velocity^39,42^. An investigation of aortic diameter and Agatston units reported increasing mean aortic diameter with increasing CAC, and demonstrated strong statistical association between >400 Agatston units and aortic diameter^34^.

We reported similar burden of elevated aortic calcification among males and females in our sample. Similar to coronary calcification scores, previous research has demonstrated consistent differences, attributing higher burden of calcification in men than women when controlling for age^43,44^. However, among MESA study participants, the majority of men and women in their sixth decade of life have observable aortic calcification^43^. While the average age of this study’s participants are younger than 50, the same MESA study found that the prevalence of elevated abdominal aortic calcification was consistent across age groups and did not differ between sexes^43^.

The MAC score was calibrated to correlate elevated coronary calcium with abdominal wall percent calcification. It is worth noting that the proportion of participants with elevated aortic calcium align more closely with the balanced MAC score than the MAC score prioritizing sensitivity. It is possible that this similarities between the elevated aortic calcium across participants could be attributed to random error, or selection bias from conditioning on abdominal CT scans. Phenotypic differences in calcium deposits, mean wall calcification percent and attenuation, was notably higher for females than males. Smaller lipid cores are typically observed in higher density atherosclerotic plaque, and higher density plaques are less vulnerable to rupture and CVD-events^8,45–47^. Further research to investigate sex-specific risk regarding phenotypic distributions of aortic calcium is warranted.

We see the MAC score as a potential mechanism to rule-in participants for further cardiovascular screening, not as one to replace implemented risk scores or coronary calcification scans. As a patient receives abdominal medical imaging, a MAC score could be calculated and reported to the physician. While atherosclerotic CVD (ASCVD) models are important and impact clinical tools, concerns regarding can include overestimation of risk, and inadequate external validity^48–51^. While CAC scores paired with ASCVD models improve clinical prognostic information, the information gain from the pairing of ASCVD scores and aortic calcification remain to be evaluated^52,53^.

Understanding the information gained between atherosclerotic CVD scores, conventional CVD assessments, and aortic calcification will be vital to future clinical implementation of aortic calcification scores. While CVD is typically detected at advanced stages or after a clinical event, atherosclerosis has an extensive subclinical phase^54,55^. While substantial evidence suggests CAC’s additive ability to predict CVD event risk in asymptomatic individuals, CAC scans are not clinically encouraged in those with traditionally low-risk profiles-$\lnot$resulting in missed identification opportunities in those susceptible, those in early stages of cardiovascular disease, or those where conventional risk assessment is limited^8,56–58^. Leveraging this subclinical phases may provide insight towards early indication of CVD burden, and early recognition could influence intervention and prevention^54,59^. Future work would ideally evaluate variance explained, or area under the curve assessments.

This study is not without limitations. First, Berkson’s bias may exist due to (1) cohort being conditioned on trauma patients, (2) the dependencies of injury and clinical need for computed tomography scans, and (3) scan availability. Further selection biases may exist based on geographical locations and socioeconomic status. As some medical records were incomplete, conservative assumptions were made to impute Framingham risk scores. As there may be selection biases that influence which participants have medical record data available, population-level imputed values were chosen rather than model based imputation procedures. A measurement bias imputation may influence the results of the study. Abdominal vertebral levels were not available for all participants due to scanning parameters. In order to reduce selection bias in scan selection, participants were not excluded based off of vertebral levels available. Finally, the Pooled Cohort Risk equations have been the the standard ASCVD prediction algorithm since its introduction in 2013^60^. While the Pooled Cohort Risk equations are typically considered to be more representative and more appropriate than the Framingham Risk Scores, data limitations prevented proper implementation^61^.

We believe this to be the first manuscript reporting granular distributions of aortic anatomy and calcification from a clinically relevant reference population. Given the heterogeneity between participants in the same population and within an individual’s anatomy, individual assessment should reflect specific measurements indexed by anatomical location and controlled for age and sex. Measuring aortic calcification opportunistically may provide effective risk assessment to inform cardiovascular risk factor modification and improve cardiovascular event surveillance efforts.

## Methods

**Figure 4:**
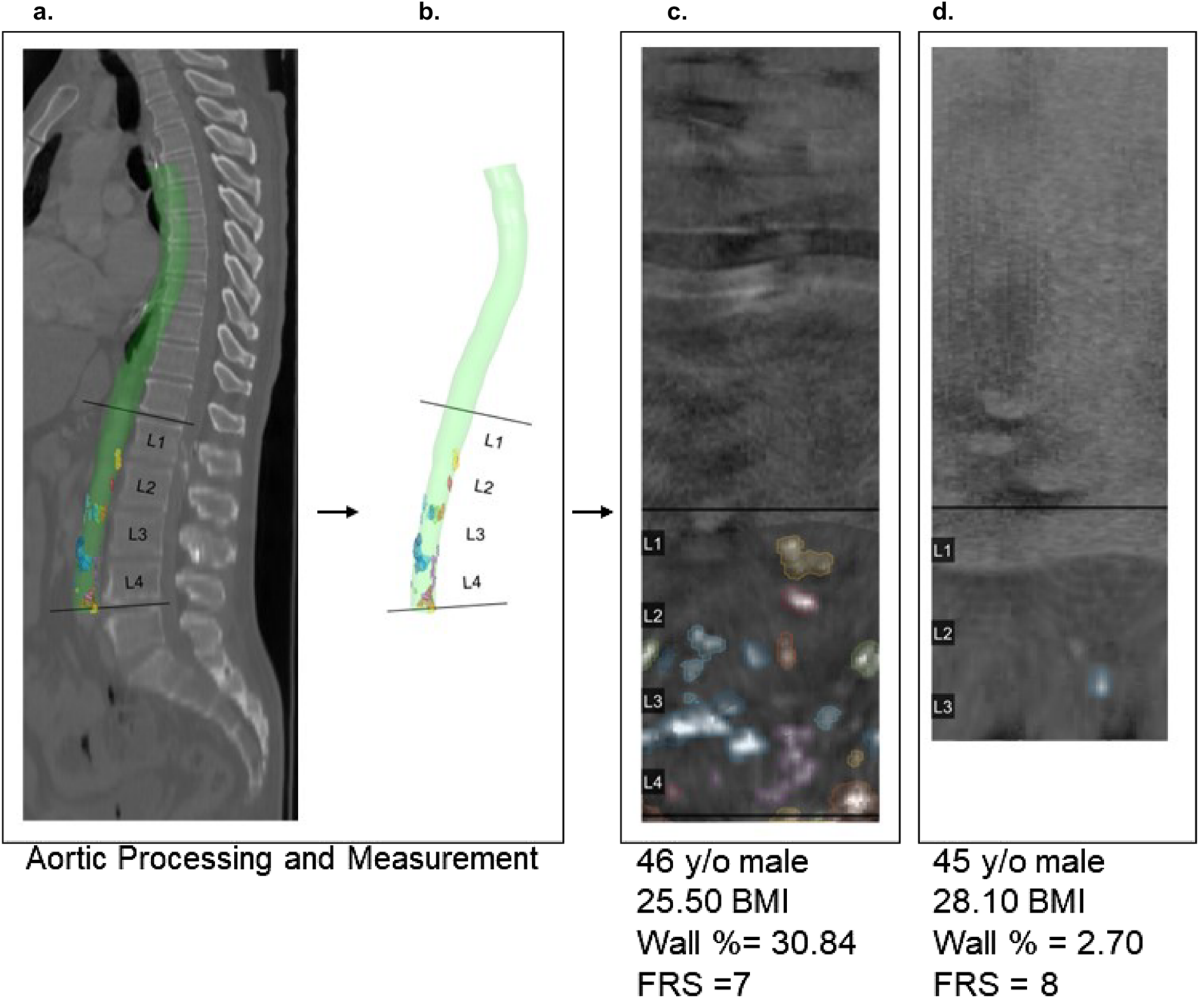
Morphomic processes for extracting aortic measurement. From left to right: (**a**) Processed aorta with calcification measurements for participant in (**c**), indexed by vertebral level, (**b**) extracted aorta, aortas for examples (**c**) and (**d**) are unfurled for visual inspection. Examples (**c**) and (**d**) are male, have relatively similar BMI and FRS but have similar BMI and are male, but (**c**) has much greater wall calcification percentage than (**d**). Note: L4 level missing for participant (**d**).

The Reference Analytic Morphomics Population has been developed to provide an approximate reference distribution of vehicle occupants in the United States of America^28,62^. Participants between the ages of 1 and 91 were scanned for trauma indications at Michigan Medicine in Ann Arbor, Michigan between January 1998 and December 2015 (N 6485)^63^. For this analysis participants without lower-level abdominal scans (n = 1155), those with stents in the abdominal aorta (n = 27), and those less than 18 years of age (n=1255) were removed from consideration (final n = 4077).

Anatomical measurements of the descending aorta were extracted using Analytic Morphomics. A central aortic lumen zone was identified on each relevant CT slice between L1 and L4 and its mean pixel value was computed for reference. Aortic calcification was determined by a dynamic threshold: calcification was identified as morphologic regions having pixel values five standard deviations above the reference^15,23,25^. Figure 4 displays a visualization of calcification region and resulting MAC scores for a higher (c.) and lower risk (d.) RAMP participant. After algorithmic measurement, CT scans were visually inspected for quality control purposes. The morphomic aortic calcification (MAC) score is the percentage of the aortic wall obfuscated by aortic calcification^15^. MAC is considered elevated when it exceeds the clinically relevant threshold at the L3 or L4 level^15^. Out of the three reported thresholds, the maximum sensitivity threshold of 4.21% was chosen to maximize the probability of discovering those with elevated atherosclerosis. The threshold balancing sensitivity and specificity was also reported. As this method could be implemented as a screening mechanism for elevated atherosclerotic burden, we valued the ability to correctly identify those with atherosclerosis rather than ruling out those with normal or absent levels.

Framingham risk scores were developed using standard algorithms^64–66^. Framingham-related variables were extracted from the medical record. Aggregated patient demographics, diagnoses, procedures, medications, and labs were extracted and merged with morphomic variables. Diagnoses were based off of International Classification of Diseases, ninth revision (ICD-9) and Charlson Comorbidity Index components, where available. Covariates were extracted from the closest encounter to the date of the CT scan. Anthropometric and morphomic variables were taken from the time of the CT Scan. The largest time frame considered was 25 years for lab-related variables. Proportion of missing values are as follows: BMI 26.54%, HDL cholesterol 74.91 %, Total Cholesterol 74.91%, systolic blood pressure 19.22%, and smoking 48.12%. Given limitations of the medical record, assumptions regarding the extracted data are as follows: 1. Participant was assumed to be a non-smoker, unless self-reported and available in the record. 2. Participant was assumed to be without diabetes unless reported in the record. 3. If the systolic blood pressure was not available, the reported score was imputed to be 0 (between 120 and 130 mmHg). 4. If total or HDL cholesterol was missing, then the score was imputed based on the BMI score. 5. If the BMI was not available, the reported BMI was imputed to be <25. 6. If blood pressure medication data was not available, the median of the two possible medication modifier scores was used. 7. Cholesterol and systolic blood pressure measurements must have been taken within 25 years of the CT scan.

Descriptive statistics are reported to examine the differences of demographics, aortic, and calcification-related variables between males and females in the RAMP sample. Linear regression was used to confirm statistical associations where appropriate. Due to the large number of participants with no observed calcification, MAC was long transformed after adding + 0.01 prior to linear regression. Due to the high number of participants with undetectable calcification area and volume, the 90th percentile was reported in lieu of the 25th and 75th percentile. Statistical analyses were performed in R 4.1^67^. All relevant plots were developed with the ggplot2 package^68^.

### Ethical approval and informed consent

This study was approved by the University of Michigan Medical School Institutional Review Board (IRBMED) (HUM-00041441). Michigan Medicine falls under the jurisdiction of IRBMED, overviewed by the Human Research Protection Program coordinated by the University of Michigan. IRBMED is granted authority to waive informed consent under certain criteria as outlined in the “Common Rule”. The United States Policy for the Protection of Human Subjects, 45 CFR part 46, outlines the criteria and mechanisms for protection of human subjects research. An independent ethics committee does not exist at the University of Michigan or Michigan Medicine. As existing CT scans were used in this study in retrospective fashion, requirement for informed consent was waived. All methods were performed in accordance with the relevant guidelines and regulations of the United States.

## Data Availability

Datasets generated during and/or analyzed during the current study are available from the corresponding author upon reasonable request.
